# Correlation Between Body Mass Index (BMI) and Postoperative Complications in Elective General Surgery: A Multicenter Study

**DOI:** 10.7759/cureus.94922

**Published:** 2025-10-19

**Authors:** Yashar Mashayekhi, Muhammad Idrees Shabbir, Sara Baba-Aissa, Uswa Abbas, Safwan Ahmad Khan, Muhammad Zeeshan Akram, Subhan Tariq

**Affiliations:** 1 Medicine, Leicester University Hospital, Leicester, GBR; 2 Surgery, Fatima Jinnah Medical University Sir Gangaram Hospital, Lahore, PAK; 3 General Internal Medicine, Leicester Royal Infirmary, Leicester, GBR; 4 Surgery, Avicenna Hospital, Lahore, PAK; 5 General Surgery, Allied Hospital, Faisalabad, PAK; 6 Orthopedics, University Hospitals Birmingham, Birmingham, GBR; 7 Trauma and Orthopedics, King Edward Medical University Lahore, Lahore, PAK; 8 Emergency Medicine, Al-Sheikh Jinnah Memorial Teaching Hospital, Sialkot, PAK

**Keywords:** bmi, complications’, patients satisfaction, surgery, surgical site infection(ssi)

## Abstract

Background: Body mass index (BMI) is a widely used measure for assessing nutritional status and has been linked to surgical outcomes. Both obesity and underweight status may predispose patients to adverse postoperative events.

Objective: This study aimed to assess the correlation between BMI and 30-day postoperative complications and to determine the association between BMI categories and the incidence, type, and severity of these complications.

Methods: This cross-sectional multicenter study was conducted at Al-Sheikh Jinnah Memorial Teaching Hospital, Sialkot; Ganga Ram Hospital, Lahore; and Avicenna Hospital, Lahore, from October 2024 to April 2025. A total of 355 adult patients (aged 18-70 years) scheduled for elective general surgical procedures were enrolled using nonprobability consecutive sampling. Preoperative BMI was calculated and categorized according to the WHO classification. Postoperative complications within 30 days were recorded and classified using the Clavien-Dindo grading system.

Results: The mean age of the study population was 44.7 ± 13.2 years, with 198 males (55.8%) and 157 females (44.2%). The mean BMI was 27.4 ± 4.9 kg/m², with 7.9% underweight, 34.9% normal weight, 35.5% overweight, and 21.7% obese. Overall, 92 patients (25.9%) developed postoperative complications, with rates highest in obese (36.4%) and underweight (25.0%) groups compared to normal-weight patients (18.5%) (p = 0.003). Surgical site infection was the most common complication (10.7%), followed by pulmonary complications (5.9%) and wound dehiscence (4.2%). Obese patients had significantly longer operative times and hospital stays.

Conclusion: Extremes of BMI, particularly obesity, are associated with higher rates of postoperative complications in elective general surgery. Preoperative BMI assessment should be integrated into surgical risk stratification, and targeted optimization strategies should be implemented to reduce BMI-related morbidity.

## Introduction

Body mass index (BMI) is one of the most widely accepted and utilized anthropometric measurements for evaluating an individual’s weight status in relation to height [1]. It is computed by dividing the body weight in kilograms by the square of the height in meters (kg/m^2^). The World Health Organization (WHO) uses it to classify people as underweight (<18.5 kg/m^2^), normal weight (18.524.9 kg/m^2^), overweight (25.029.9 kg/m^2^), and obese (220.0 kg/m^2^) [2]. Although BMI is a relatively crude instrument that does not differentiate between lean mass and fat mass, it is still a handy tool in clinical risk stratification. The deviations of the normal BMI scale have been associated with numerous physiological changes, metabolic disorders, and systemic situations that may either directly or indirectly affect the outcomes of the surgical process. Both curves of BMI may introduce special difficulties in surgical practice [3]. The effects of underweight patients include sparse protein reserves, hypoalbuminemia, and poor immunity, which can negatively affect the wound healing process and lead either to contracting an infection or persistent recovery. On the contrary, the overweight and obese patients might possess greater adipose tissue, making access to areas requiring surgery difficult, long operative time, and increased blood loss during surgery [4]. Additionally, comorbid conditions are often linked to obesity, and these include type 2 diabetes mellitus, hypertension, coronary artery disease, chronic respiratory disease, and metabolic syndrome. Such conditions may increase the perioperative risk and the difficulty of anesthetic management, which eventually may affect the postoperative recovery and complication profile [5].

This area of elective general surgery is wide and covers procedures done under nonemergency conditions, including laparoscopic cholecystectomy, inguinal and incisional hernia repair, thyroidectomy, and colorectal resections. The fact that these operations are elective gives us a chance to perform adequate preoperative assessment/optimization of comorbid conditions. Nevertheless, the presence of postoperative complications is still a serious medical issue [6]. Such complications can comprise the surgical site infection, the wound dehiscence, the formation of a seroma or a hematoma, the pulmonary complications (e.g., atelectasis, pneumonia), the occurrence of a thromboembolic event, the anastomotic leakage, and the cardiovascular events. The interplay that exists between BMI and postoperative complications can only be described as multifaceted and continues to attract a unique debate [7]. Multiple studies have established a positive correlation between unfavorable surgical outcomes and high BMI, which can be explained by the presence of technical challenges during surgery, poor perfusion of tissues, impaired inflammatory responses, and postoperative limited mobility. As an illustration, the wound infection rates have been reported to be higher in the case of obesity, which is explained through the increased number of skin folds, thicker bacterial colonization, and decreased adipose tissue oxygen tension. At the opposite spectrum, malnourishment and low BMI have been associated with no-collagen synthesis, faulty tissue adherence, and ineffective immunity defense mechanisms that can likewise result in the compromise and systemic infections [8].

Indicatively, certain studies have suggested a U-shaped correlation, according to which both categories of BMIs, low and high, come with increased risks as compared to people with normal BMI. Such an apparent protective effect of mild obesity (or the so-called obesity paradox) has been noted in some groups of surgical patients and in patients receiving critical care [9]. The prolonged effects of being overweight are, however, undesirable, especially in terms of cardiovascular risks and metabolic diseases. Fluctuation in the results of studies could be explained by a variation in the patient population, the surgical specialties, the perioperative protocol, and complications' definition [10]. As an example, the impact of BMI can vary between minimally invasive technical and open procedures, between clean and dirty surgeries. Additionally, the possible confounding factors of this association between BMI and outcomes include the presence of comorbid conditions, age, smoking status, and the preoperative optimization of patient nutrition [11]. This relation is even more clinically relevant in countries with low- and middle-income economies since they experience the so-called double burden of malnutrition where both undernourishment and obesity occur simultaneously [12].

Objective

This study aimed to assess the correlation between BMI and 30-day postoperative complications and to determine the association between BMI categories and the incidence, type, and severity of these complications.

## Materials and methods

This cross-sectional multicenter study was conducted at Al-Sheikh Jinnah Memorial Teaching Hospital, Sialkot, Ganga Ram Hospital, Lahore, and Avicenna Hospital, Lahore, from October 2024 to April 2025. A total of 355 patients scheduled for elective general surgery were included in the study. The sample size was based on recruitment feasibility, not on an a priori calculation. Patients were recruited using a nonprobability consecutive sampling method. A uniform perioperative protocol was applied across both participating centers to minimize variability in anesthetic management, intraoperative care, and postoperative monitoring. Adult patients aged between 18 and 70 years who were scheduled for elective general surgical procedures and classified as American Society of Anesthesiologists (ASA) physical status I to III were included. All participants provided written informed consent before enrollment. Patients undergoing emergency surgeries, those with incomplete or missing medical records, individuals with pre-existing systemic infections or active malignancy, and those undergoing palliative procedures were excluded from the study.

Data collection procedure

After taking approval from the institutional review board and ethical committee, eligible patients were approached for participation. Demographic and clinical information was captured by means of a structured data collection proforma. BMI was computed as the weight of the patients in kilograms divided by square meter (kg/m^2^) and classified into the following WHO categories: underweight (<18.5), normal weight (18.5 to 24.9), overweight (25 to 29.9), and obese (30 or above). The general anesthesia and perioperative care patterns were standardized during all surgeries. Postoperative complications were monitored during hospital stay and for 30 days after surgery, as this period is internationally recognized for capturing surgery-related morbidity (American College of Surgeons-National Surgical Quality Improvement Program (ACS-NSQIP) standards). The complications that were recorded include surgical site infection, wound dehiscence, seroma, hematoma, lung-related complications, i.e., atelectasis or pneumonia, cardiovascular complications, i.e., arrhythmia or myocardial infarction, thromboembolism, and urinary tract infections. All complications have been categorized into the Clavien-Dindo grading system in order to offer a uniformity of measurements of severity. Postoperative complications were noted by research nurses and staff.

Statistical analysis

Data were analyzed using IBM SPSS Statistics for Windows, Version 26 (Released 2018; IBM Corp., Armonk, New York, United States). Continuous variables (age, BMI, operative time, hospital stay) were presented as mean ± SD, and categorical variables (gender, BMI category, complications) as frequencies and percentages. Group comparisons were made using analysis of variance (ANOVA) for continuous data and Chi-square or Fisher’s exact test for categorical data. Variables significant at p < 0.10 in univariate analysis were entered into multivariate logistic regression to identify independent predictors of complications. Odds ratios (ORs) with 95% confidence intervals (CIs) were reported, and a p-value ≤ 0.05 was considered statistically significant.

## Results

Data were collected from 355 patients. The mean age was 44.7 ± 13.2 years, with a slight male predominance (55.8%). The average BMI was 27.4 ± 4.9 kg/m², with overweight (35.5%) and normal weight (34.9%) being the most common categories, followed by obese (21.7%) and underweight (7.9%). The most frequent procedures performed were laparoscopic cholecystectomy (32.1%), hernia repair (27.0%), and colorectal surgery (18.6%). The mean operative time was 92.4 ± 21.7 minutes, and the mean hospital stay was 4.3 ± 2.1 days (Table 1).

**Table 1:** Baseline demographic and clinical characteristics of study participants (N = 355)

Characteristic	Total (N = 355)
Age (years), mean ± SD	44.7 ± 13.2
Gender, n (%)	
Male	198 (55.8)
Female	157 (44.2)
BMI (kg/m²), mean ± SD	27.4 ± 4.9
BMI category, n (%)	
Underweight (<18.5)	28 (7.9)
Normal weight (18.5-24.9)	124 (34.9)
Overweight (25.0-29.9)	126 (35.5)
Obese (≥30.0)	77 (21.7)
Common procedures, n (%)	
Laparoscopic cholecystectomy	114 (32.1)
Hernia repair	96 (27.0)
Colorectal surgery	66 (18.6)
Others	79 (22.3)
Operative time (min), mean ± SD	92.4 ± 21.7
Hospital stay (days), mean ± SD	4.3 ± 2.1

Overall postoperative complication rates

Overall, 25.9% of patients developed complications, with the highest rate in obese patients (36.4%) and the lowest in normal-weight patients (18.5%), a statistically significant difference (p = 0.003). Surgical site infection was the most common complication, occurring in 18.2% of obese patients compared to 7.3% of normal-weight patients (p = 0.021) (Table 2).

**Table 2:** Postoperative complications by BMI category (N = 355) BMI: body mass index

Complication	Underweight (n = 28)	Normal (n = 124)	Overweight (n = 126)	Obese (n = 77)	χ² statistic	p-value
Any complication, n (%)	7 (25.0)	23 (18.5)	33 (26.2)	28 (36.4)	13.72	0.003
Surgical site infection	3 (10.7)	9 (7.3)	13 (10.3)	14 (18.2)	9.68	0.021
Pulmonary complication	1 (3.6)	5 (4.0)	7 (5.6)	8 (10.4)	7.91	0.048
Wound dehiscence	1 (3.6)	3 (2.4)	5 (4.0)	6 (7.8)	8.34	0.039
Cardiovascular event	0 (0.0)	2 (1.6)	3 (2.4)	3 (3.9)	3.28	0.187
Thromboembolism	0 (0.0)	1 (0.8)	2 (1.6)	3 (3.9)	5.14	0.121

Type of Complications by BMI Category

**Figure 1:**
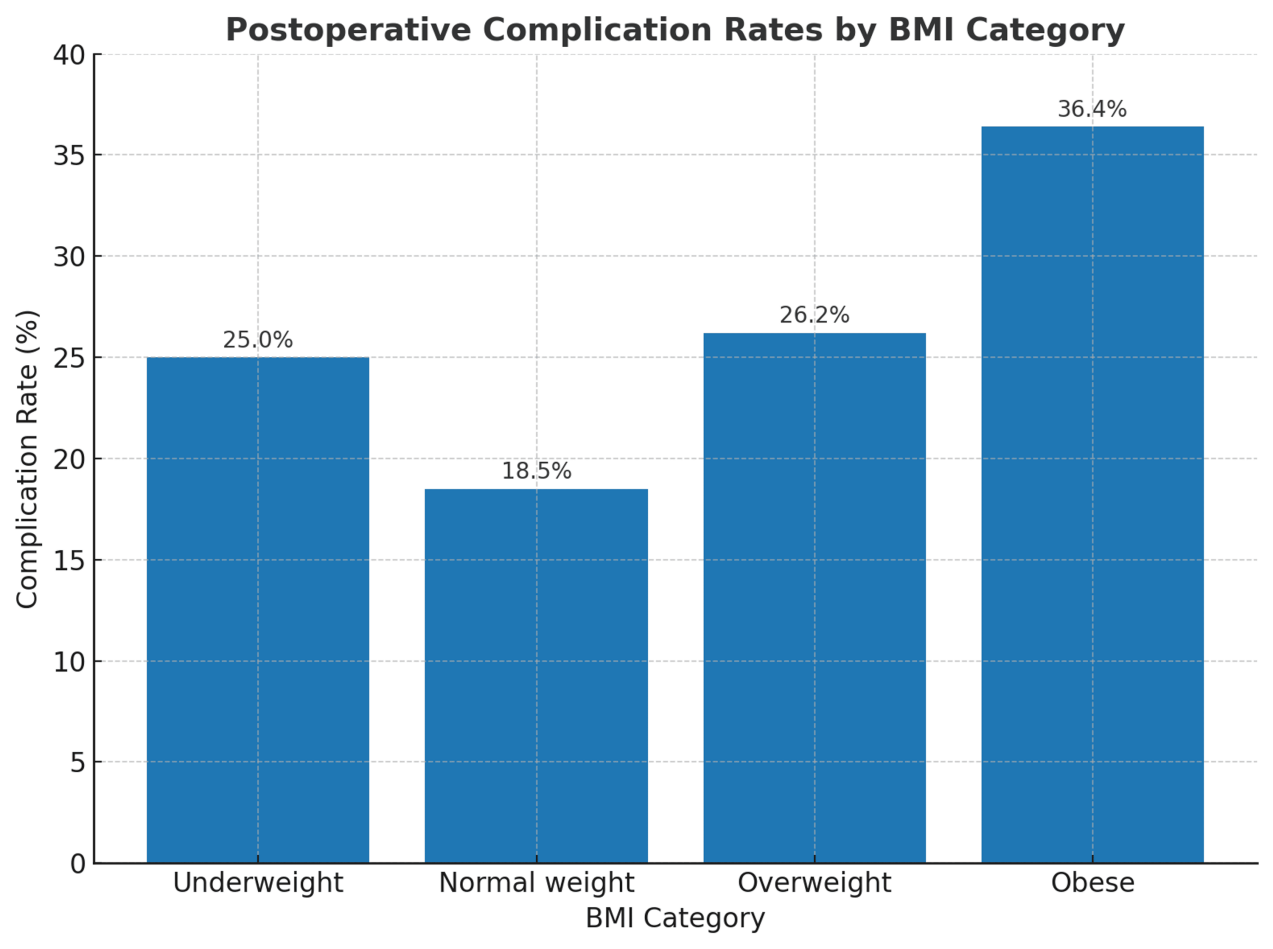
Postoperative complication rates according to BMI category BMI: body mass index

Most complications were Grades I and II, indicating mild to moderate severity requiring minimal intervention or pharmacological treatment. Obese patients had the highest proportion of Grade II (16.9%) and Grade III (7.8%) complications. Severe complications (Grade IV) were rare, occurring in 2.0% of the cohort, with no Grade V (mortality) events recorded (Table 3, Figure 1).

**Table 3:** Distribution of postoperative complication severity by Clavien-Dindo classification (N = 355)

Clavien-Dindo Grade	Underweight (n = 28)	Normal (n = 124)	Overweight (n = 126)	Obese (n = 77)	Total (N = 355)	χ² statistic	p-value
Grade I (minor, no intervention)	3 (10.7)	8 (6.5)	9 (7.1)	8 (10.4)	28 (7.9)	2.81	0.421
Grade II (pharmacological treatment)	3 (10.7)	9 (7.3)	14 (11.1)	13 (16.9)	39 (11.0)	6.41	0.093
Grade III (surgical/endoscopic)	1 (3.6)	4 (3.2)	8 (6.3)	6 (7.8)	19 (5.4)	4.86	0.286
Grade IV (life-threatening complication)	0 (0.0)	2 (1.6)	2 (1.6)	3 (3.9)	7 (2.0)	3.05	0.382
Grade V (death)	0 (0.0)	0 (0.0)	0 (0.0)	0 (0.0)	0 (0.0)	-	-

Severity of Complications (Clavien-Dindo Classification)

Obese patients had the longest operative times (99.7 ± 24.5 minutes) and hospital stays (4.9 ± 2.4 days), followed by overweight patients, with both differences reaching statistical significance for time (p = 0.012) and stay (p = 0.046) (Table 4).

**Table 4:** Mean operative time and hospital stay by BMI category BMI: body mass index

BMI category	Operative time (min), mean ± SD	Hospital stay (days), mean ± SD	F-statistic (time)	p-value (time)	F-statistic (stay)	p-value (stay)
Underweight (<18.5)	85.6 ± 19.3	4.5 ± 2.0	4.32	0.012	3.01	0.046
Normal weight (18.5-24.9)	88.2 ± 18.7	3.9 ± 1.8				
Overweight (25.0-29.9)	94.8 ± 22.1	4.4 ± 2.2				
Obese (≥30.0)	99.7 ± 24.5	4.9 ± 2.4				

## Discussion

The present study investigated the correlation between BMI and postoperative complications in patients undergoing elective general surgery, involving a cohort of 355 individuals categorized according to the WHO BMI classification. Higher BMI was found to be significantly linked to an increased risk of postoperative complications, particularly pulmonary complications and surgical site infections (SSI). Obese patients (BMI ≥30 kg/m²) exhibited the highest overall complication rate (36.4%), followed by overweight patients (26.2%), while normal-weight individuals had the lowest rate (18.5%). Interestingly, underweight patients also experienced relatively high complication rates (25.0%), suggesting that both extremes of BMI may confer increased surgical risk. Obesity is a known risk factor for postoperative morbidity in general surgery, as demonstrated by our findings and previous research. Studies have consistently reported higher rates of wound complications in obese individuals due to factors such as excessive adipose tissue, reduced vascularity, and impaired oxygen diffusion, all of which contribute to delayed healing and increased susceptibility to infection [13]. Furthermore, technical challenges during surgery in obese patients, such as difficulty in exposure, longer operative time, and increased blood loss, may contribute to poorer outcomes. This link between BMI and surgical complexity is bolstered by the current study's finding that obese patients had significantly longer operative times (99.7 to 24.5 minutes) than normal-weight patients (88.2 to 18.7 minutes). In this study, obese patients also had a higher rate of pulmonary complications. This was probably because obese patients had lower lung compliance, a higher rate of obstructive sleep apnea, and a higher risk of hypoventilation during surgery [14]. In large-scale database studies, obesity was linked to an increased risk of postoperative pneumonia and respiratory failure. Similar findings have been found. The increased hospital stay observed among obese individuals in our cohort further reflects the additional recovery time often required to manage such complications [15].

Despite their smaller absolute numbers, underweight patients on the other end of the BMI spectrum also demonstrated a significant complication burden. This may be explained by poor nutritional status, reduced protein reserves, and impaired immune function in this population, leading to decreased wound tensile strength and slower healing. Low BMI is independently linked to higher postoperative mortality and morbidity, particularly in gastrointestinal and oncologic surgeries, according to several studies. Even in nonemergency elective settings, these findings emphasize the significance of recognizing malnutrition as a risk factor for surgery [16]. Interestingly, the “obesity paradox,” the notion that overweight or mildly obese patients may have equal or better short-term postoperative survival compared to normal-weight individuals, was not strongly supported by our data. While overweight patients had slightly lower complication rates than the obese group, their rates remained higher than those of normal-weight patients. This suggests that any potential protective effect of increased BMI may not be as relevant for complication risk, even if survival outcomes differ [17]. The Clavien-Dindo classification of the distribution of complication severity revealed that the majority of complications were of mild to moderate severity (Grades I-II), necessitating pharmacologic treatment or minor interventions. However, severe complications (Grades III-IV) occurred more frequently in overweight and obese groups, emphasizing the importance of BMI as a determinant not only of complication occurrence but also of complication severity [18]. These findings highlight the importance of implementing targeted perioperative optimization strategies for patients at both extremes of BMI. For obese individuals, preoperative interventions such as weight reduction programs, improved glycemic control, and respiratory prehabilitation can help minimize risk. For underweight patients, addressing micronutrient deficiencies and ensuring adequate nutritional support may enhance recovery. Additionally, meticulous surgical technique, efficient intraoperative planning, and the use of minimally invasive approaches whenever feasible can further mitigate BMI-related complications.

Limitations

This study has certain limitations that should be considered when interpreting the results. The use of nonprobability consecutive sampling may introduce selection bias and limit external validity. Although perioperative protocols were standardized, variations in surgical technique and provider experience could have influenced complication rates. Potential confounders such as smoking status, comorbidities, nutritional markers, and preoperative functional status were not fully adjusted for, which may have affected the observed associations. Additionally, the absence of long-term follow-up limits the assessment of late postoperative outcomes. Future studies with randomized designs and multivariate adjustment are recommended to validate these findings.

## Conclusions

It is concluded that both extremes of BMI obesity and underweight status are associated with an increased risk of postoperative complications in elective general surgery, with obesity showing the highest overall complication rates, particularly for surgical site infections and pulmonary events. Normal-weight patients demonstrated the lowest complication burden, underscoring the value of optimal preoperative nutritional and weight status. These findings highlight the importance of incorporating BMI assessment into preoperative risk stratification and counseling, while also implementing targeted perioperative optimization strategies such as nutritional support for underweight patients and weight management interventions for obese individuals.
